# Impact of autologous HSCT on the quality of life and fatigue in patients with relapsing multiple sclerosis

**DOI:** 10.1038/s41598-022-19748-7

**Published:** 2022-09-13

**Authors:** N. Giedraitiene, G. Gasciauskaite, G. Kaubrys

**Affiliations:** 1 Clinic of Neurology and Neurosurgery, Institute of Clinical Medicine, Faculty of Medicine, Vilnius University, Vilnius, Lithuania; 2 Institute of Anaesthesiology, University Hospital Zurich, Zurich, Switzerland

**Keywords:** Neurology, Neurological disorders, Multiple sclerosis

## Abstract

In this study, we aimed to assess the quality of life, fatigue, anxiety, and depression after Autologous haematopoietic stem cell transplantation (AHSCT) and to investigate its impact of on separate domains of health status and fatigue in patients with multiple sclerosis (MS). Overall, 18 patients with highly active relapsing MS (mean age 36.3 years, 83.3% female) underwent the AHSCT in Vilnius Multiple Sclerosis center, and we prospectively collected Short Form 36, Health Survey Questionnaire, Fatigue Descriptive Scale, and Hospital Anxiety and Depression Scale beforeand Month3, 12, and 24 after AHSCT. The median score of Expanded Disability Status Scale at Month3 after transplant improved in 14 patients (77.8%). A significant improvement in physical functioning, vitality, and pain was found at Month3 after AHSCT (*p* < 0.05), which was sustained until Month12 and 24. The improvement in fatigue score was found at Month12 after AHSCT, which was sustained until Month24. Decrease in EDSS score had a positive impact on the better HRQoL outcomes, especially physical and social outcomes. Thus, AHSCT improved quality of life and reduced symptoms of fatigue in patients with highly active relapsing MS. The improvement was determined earlier in the domains of QoL than in the fatigue.

## Introduction

Multiple sclerosis (MS) is a potentially disabling disease that can cause physical, emotional, and cognitive impairment. Immune ablation followed by autologous hematopoietic stem cell transplantation (AHSCT) has proven to be an effective long term treatment for younger patients with highly active relapsing MS showing clinical and radiological evidence of high disease activity^1,2^. The results obtained from different studies indicate that up to 86.9% of patients with MS who received AHSCT remained relapse-free, up to 91.3% were EDSS progression-free and 86.3%—MRI event-free^3,4^.

MS significantly affects patients’ quality of life (QoL), interfering with their ability to work, pursue leisurely activities, and execute daily life tasks^5,6^. To understand how MS impairs patients’ QoL, the psychological aspects of the disease along with physical aspects and disability should be considered. Several studies have investigated QoL in patients with MS and its’ results vary across regions, cultures and health care systems^7,8^. Physicians have commonly singled out physical symptoms as the most important negative aspect of this illness, equating health to absence or reduction of disease, and not to complete physical, mental, and social well-being^9^. However, MS relapses, disability progression and magnetic resonance imaging that reflect the disease activity comprise only part of the impact that MS has on a patient’s daily life^10^. In recent years, scientists have been recommending the evaluation of health-related quality of life (HRQoL) be included in the definition of No Evidence of Disease Activity^11^. In line with this, previous studies have shown that patients and physicians disagree on which health domains are most important in MS and its influence on daily life^8^.

Physical disability only partly impairs QoL in MS. MS-associated fatigue and depression are common and treatable features of the disease, which could also impact on QoL, independent of physical disability^12^. Fatigue in MS affects up to 90% of patients, and at least one half of them report it as one of their worst symptoms^12,13^. The pathophysiological causes of the fatigue vary. Mostly, the primary cause of fatigue in MS is the inflammation in the central nervous system^12–14^, whereas the secondary cause may include fatigue from muscle weakness. Depression, anxiety, and increased attemps in daily activities owing to physical disability also play a role in fatigue^15,16^. However, fatigue is subjective and varies over the course and severity of the disease, which makes its measurement challenging.

The incidence of psychopathological disorders, especially depression and anxiety, is high in MS, often, higher than in other chronic neurological disorders^17,18^. Clinical studies suggest that more than half of the patients with MS have symptoms of depression and anxiety, which negatively impacts all HRQoL domains. The depressive symptoms in MS also correlate with fatigue^19,20^.

Treatment targeting HRQoL and fatique in MS have had mixed levels of success. Some treatments aim to address comorbidities that might contribute to MS such as depression, anxiety, and spasticity or pain treatment through various pharmacologic mechanisms. There is also data showing that disease modification thorough immune modulation or suppression can improve fatigue, reduce anxiety, and improve QoL in patients with MS. During the last 30 years, autologous hematopoetic stem cell transplantation (AHSCT) was proposed as a treatment for severe autoimmune diseases and is a promising alternative treatment for highly active MS^21,22^.

Since AHSCT is an innovative therapy in the treatment of highly active MS, there is an urgent need for studies which can assess not only the effectiveness and safety of the treatment, but also its impact on the patients' QoL, fatigue, anxiety and depression. Further data is being provided regarding AHSCT in MS treatment; however, despite the wide recognition of the importance of evaluating the emotional aspects, most studies apply the clinical approach.

The study aimed to assess the QoL, fatigue, anxiety and depression after AHSCT in patients with MS and to investigate the impact of AHSCT on the separate domains of health status and fatigue in these patients after AHSCT.

## Results

### Patients

Eighteen patients treated with AHSCT were included in the study. HRQoL, depression, anxiety and fatigue were assessed in these patients before AHSCT, and thereafter, at Month 3, 12 and 24. Demographic and clinical characteristics of the patients at baseline are provided in Table 1.

**Table 1:** Numerical and percentage distribution of patients with MS undergoing AHSCT, according to demographic and clinical characteristics.

Demographic and clinical variables	N	%
Female/male	15/3	83.3%/16.7%
Mean age and SD at the beginning of the study (years)	35.2 ± 9.1	–
Mean time of education and SD (years)	16.3 ± 3.4	–
Disease duration (years)	8.6 ± 5.5	
Median EDSS score before AHSCT (IQR)	6.0 (6.0–6.5)	
**Previous DMT**		
Fingolimod	8	
Natalizumab	8	
Alemtuzumab	1	
Interferon-beta	1	

*AHSCT* Autologous Haematopoietic Stem Cell Transplantation, *EDSS* Expanded Disability Status Scale, *SD* standard deviation, *IQR* interquartile range, *DMT* disease modifying treatment.

Two patients of 18 (11.1%) had one relapse and one patient of 18 patients (5.6%) had two relapses during the first year after AHSCT. One patient of 18 (5.6%) had one relapse and one patient of 18 (5.6%) had two relapses during the second year after AHSCT. The annualized relapse rate dropped from 2.7 before AHSCT to 0.17 in the first year and to 0.11 in the senond year after AHSCT.

**Figure 1:**
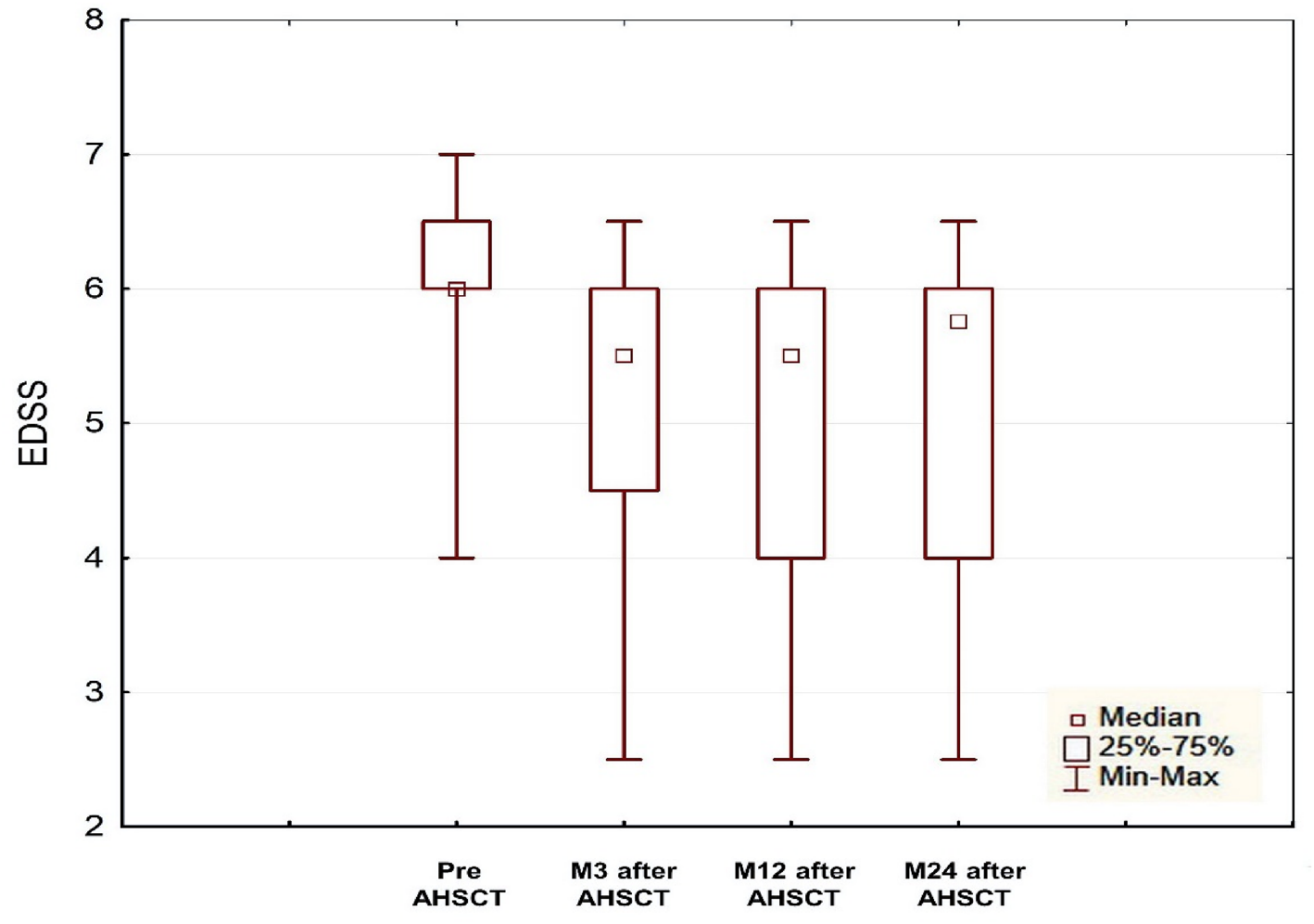
Changes in EDSS score from baseline to Month24 after AHSCT. *EDSS* Expanded Disability Status Scale, *AHSCT* autologous hematopoietic stem cell transplantation, *preAHSCT* before autologous hematopoietic stem cell transplantation, *M3* month 3, *M12* month 12, *M24* month 24.

Comparing the mean score on the Expanded Disability Status Scale (EDSS) at the four time points (preAHSCT, M3, M12 and M24), significant improvement was assessed at Month3 after AHSCT (*p* < 0.05) and EDSS score remained stable at Months 12 and 24 (Fig. 1). The median EDSS score at Month 3 with a minimal change from baseline of 0.5 points improved after transplant in 77.8% of patients; however, in 22.8% of patients EDSS, did not change. Sustained disability improvement was found in 12 (66.7%) of 18 patients at Month 12 and in 11 (61.1%) of 18 patients at Month 24.

**Figure 2:**
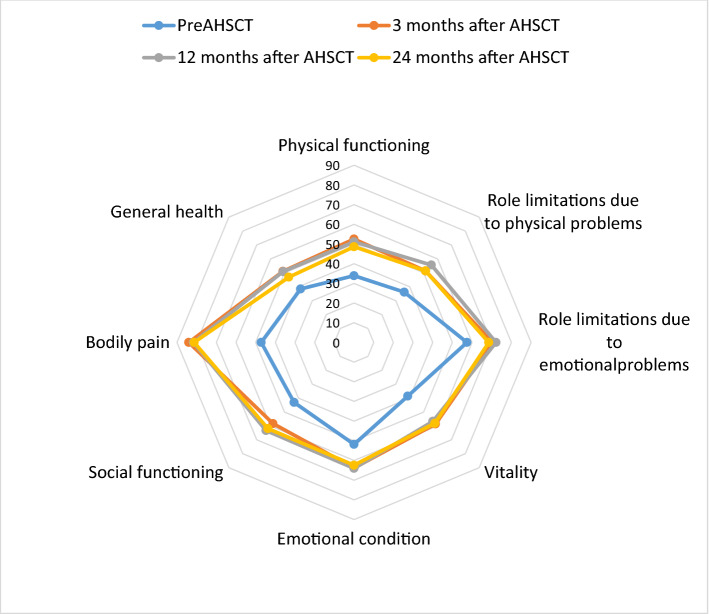
HRQoL in patients with MS before and at Month 3, Month 12 and Month 24 after AHSCT. Scored on a 0–100 scale (0 = low HRQoL; 100 = high QoL). *AHSCT* autologous haematopoietic stem cell transplantation, *PreAHSCT* before autologous haematopoietic stemm cell transplantion, *HRQoL* health-related quality of life.

An improvement in all domains of Short Form 36 (SF-36) Health Survey Questionnaire was observed at the first follow-up, that is, Month3 after AHSCT. The improvement was sustained until Month 12 and 24 (Fig. 2).

A significant improvement was observed in physical functioning, vitality, and pain at Month 3 after AHSCT (*p* < 0.05) (Table 2). The median physical functioning score improved by 18.4, vitality by 20 and pain by 36.7 at first follow-up after AHSCT (*p* < 0.05), which was sustained until the end of the study. In 22.8% of patients, who did not show improvement in EDSS score, the scores of physical functioning and vitality did not change after AHSCT. In this patients, the significant improvement in pain score was observed at Month12 after AHSCT (*p* < 0.05): the mean score improved by 51.8 at Month 12 compared with the baseline.

**Table 2:** The scores of SF-36 domains before AHSCT and 2 years after AHSCT in patients with MS.

Test	M0	M3	M12	M24	rmANOVA	Post-hoc
Physical functioning	34.1 ± 18.5	52.5 ± 22.0	51.1 ± 23.4	48.3 ± 25.9	F = 6.91; ***p* < 0.05**	**M0 < M3, M12, M24** M3 = M12 = M24
Role limitations due to physical problems	36.1 ± 37.6	51.4 ± 44.1	55.6 ± 43.3	51.4 ± 46.6	F = 0.86; *p* > 0.05	M0 = M3 = M12 = M24
Role limitations due to emotional problems	424.1 ± 1558.6	70.4 ± 39.4	72.2 ± 40.0	68.5 ± 38.7	F = 0.53; *p* > 0.05	M0 = M3 = M12 = M24
Vitality	38.6 ± 12.8	58.6 ± 16.1	56.7 ± 17.7	58.1 ± 19.9	F = 15.6; ***p* < 0.001**	**M0 < M3, M12, M24** M3 = M12 = M24
Emotional condition	51.8 ± 6.2	63.8 ± 21.0	64.0 ± 18.0	62.4 ± 18.8	F = 2.0; *p* > 0.05	M0 = M3 = M12 = M24
Social functioning	43.1 ± 29.1	58.3 ± 29.2	63.2 ± 27.6	61.9 ± 30.1	F = 2.6; *p* > 0.05	M0 = M3 = M12 = M24
Pain	47.2 ± 8.3	83.9 ± 20.1	81.7 ± 22.8	81.3 ± 19.6	F = 20.6; ***p* < 0.001**	**M0 < M3, M12, M24** M3 = M12 = M24
General Health	47.2 ± 19.5	51.1 ± 19.6	51.5 ± 18.5	46.9 ± 20.4	F = 0.9; *p* > 0.05	M0 = M3 = M12 = M24

rmANOVA—Repeated measures analysis of variance, AHSCT—autologous hematopoietic stem cell transplantation, M0—before AHSCT, M3—month 3 after AHSCT, M12—month 12 after AHSCT, M24—month 24 after AHSCT.

Significant values are in [bold].

9 patients (50.0%) had moderate and 7 patients (38.9%) had severe depression before AHSCT. 13 patients (72.2%) had moderate anxiety and 1 patient (5.6%) had severe anxiety before AHSCT.

Unlike the scores on the HRQoL questionnaires, where significant improvement was found at Month 3, the improvement in fatigue score was found at Month 12 after AHSCT, and this improvement was sustained until Month 24. The improvement in fatigue score was also observed at Month3; however, it was not significant (*p* > 0.05). The anxiety and depression scores were stable before AHSCT and at Month 3, 12, and 24 after AHSCT (Table 3). In 22.8% of patients, who did not have improvement in their EDSS score, their score on fatigue and anxiety also did not change after AHSCT. However, there was significant improvement in depression score in these patients at Month 12 after AHSCT (*p* < 0.05): the mean score decreased by 7.25 at Month 12 compared with the baseline.

**Table 3:** The scores of fatigue, anxiety and depression before AHSCT to 2 years after AHSCT in patients with MS.

Test	M0	M3	M12	M24	rmANOVA	Post-hoc
Fatigue	7.9 ± 5.8	4.6 ± 4.2	2.8 ± 3.8	3.0 ± 3.7	F = 7.12; ***p* < 0.01**	**M0 > M12**, M24 M0 = M3, M12 = M24
Anxiety	11.3 ± 2.1	12.2 ± 3.1	12.1 ± 2.8	11.8 ± 2.2	F = 0.83; *p* > 0.05	M0 = M3 = M12 = M24
Depression	8.3 ± 2.2	7.8 ± 2.0	7.9 ± 1.9	8.4 ± 2.6	F = 0.40; *p* > 0.05	M0 = M3 = M12 = M24

rmANOVA—Repeated measures analysis of variance, AHSCT—autologous hematopoietic stem cell transplantation, M0—before AHSCT, M3—month 3 after AHSCT, M12—month 12 after AHSCT, M24—month 24 after AHSCT.

Significant values are in [bold].

### Analysis of variables that explained health status in patients with MS during the last assessment after AHSCT

The General Linear Model was used to assess the impact of demographic and disease characteristics, anxiety, depression, and fatigue during the last visit on the separate domains of health status before and after AHSCT in patients with MS. Dependent variables in the models were the scores of SF-36 domains at Month 24. Independent variables (regressors) in the models were age, gender, disease duration, as well as the EDSS, anxiety, depression, and fatigue scores at Month 24.

Regression model that had explained separate domains of health status after AHSCT:

1. Physical functioning _M24_ = 64.1 − 17.9 × EDSS_M24_ + 4.1 × depression _M24_; R^2^ = 0.76; *p* < 0.05.
2. Role limitations due to physical problems _M24_ = 80.9 − 30.2 × EDSS_M24_; R^2^ = 0.49; *p* < 0.05.
3. Emotion conditional _M24_ = 28.9 + 5.8 × anxiety _M24_; R^2^ = 0.51; *p* < 0.05.
4. Social functioning _M24_ = − 19.4 − 13.1 × EDSS_M24_ + 5.8 × depression _M24_ + 7.9 × anxiety _M24_; R^2^ = 0.57; *p* < 0.05.

Only significant independent variables (*p* < 0.05) were included in the models.

## Discussion

The primary aim of the study was to assess the QoL, fatigue, anxiety, and depression in patients with MS after AHSCT. Since 1997, a number of trials on AHSCT in patients with MS and its efficacy and safety have been reported^21,22^. However, only two trials provide their data according to QoL^23,24^ and one trial on fatigue^25^ in patients with MS after AHSCT. In this study, we analysed the HRQoL and fatigue owing to MS by following up with 18 patients with MS on whom AHSCT was performed in one center. Moreover, we emphasized, on the impact of AHSCT on health status and fatigue in patients with MS post transplant.

Evidence from multiple studies indicates that AHSCT is a highly effective treatment alternative for highly active relapsing MS^26–28^. The current analysis shows that in patients with relapsing MS who do not respond to prior high efficacy DMT adequately, AHSCT provides disability stabilization as well as early disability regression^26,29^. Our results support previous published data, showing the ability of AHSCT to reduce the disability and to sustain the disability stability for long time^26–28^.

MS is a chronic and potentially disabling disease that significantly affects patients’ QoL. In MS, QoL is impaired partly owing to physical disability, and partly due to MS-associated fatigue, and depression. Mostly, patients with MS require life-long treatments that can cause major side-effects. Assessing the impact of these interventions on QoL and fatigue is important because it highlights the patient’s perspective on the overall effect of the treatment. Our results show that AHSCT improves HRQoL and fatigue in people with highly active relapsing MS. Absolute scores of all SF-36 domains increased at Month 3 after AHSCT and remained stable at Month 12 and 24, whereas the SF-36 domains physical functioning, vitality and pain were significantly affected by the treatment. Unlike other studies that showed significant improvement in all domains of SF-36 one year after AHSCT^6,23,24^, we found earlier improvement in HRQoL in patients MS. The lack of significance in other domains, like general health, role limitation because of physical and emotional problems, and social functioning, may be related to a relatively small sample size. In contrast to the scores of HRQoL questionnaires, the improvement in fatigue score was found later, and significant improvement was observed at Month 12 after AHSCT, which sustained until Month 24. However, non-significant decline in anxiety score was observed at Month 3, which indicates increased anxiety in patients in the early phase after transplant. Slight decline in anxiety and later improvement in fatigue score suggest that AHSCT using intermediate intensity lymphoablative conditioning regimen may negatively impact anxiety and fatigue in patients with MS in early phases after the AHSCT procedure. Previously published data have proved the negative impact of AHSCT on cognition in the early phase after AHSCT (month 2 and 3 after AHSCT)^29–31^; however, other comorbidities like fatigue and anxiety in MS are likely to be affected in the early phase after transplantation.

In almost one quarter of patients who showed no improvement in physical disability, the fatigue and anxiety scores also did not improve after AHSCT either. However, we found significant improvement in depression scores at Month 12 after transplant. Our data support that AHSCT has benefits on the disease stability and even can improve other comorbidities.

We also found an association between lower EDSS and improvement in physical and social functioning as well as role limitations owing to physical problems, during the last follow-up after AHSCT. The current analysis shows that in patients with highly active relapsing MS, disability regression is the main value that has a positive impact on HRQoL outcomes, especially physical and social outcomes. We did not find the value that could have a positive impact on fatigue scores after AHSCT; however, previous studies have demonstrated that better social outcomes are associated with reduction in fatigue score^25^.

The main limitation of this study was the relatively low sample size. Therefore, the results of this study cannot be generalized; further research is needed to replicate these findings. Furthermore, there was no comparative control group and the assessment was performed in one center. However, we did not identify any controlled study, with the exception of one small randomised controlled trial^32^ in which a comparative group is used to assess the results of AHSCT.

## Conclusions

AHSCT improved quality of life and reduced symptoms of fatigue in patients with highly active relapsing MS. The improvement was determined earlier in the domains of QoL than in the fatigue. A significant improvement in physical functioning, vitality, and pain was found at Month 3 after AHSCT (*p* < 0.05) in patients with MS, and this improvement was sustained until Month 12 and 24. The improvement in fatigue score was found at Month 12 after AHSCT, which was sustained until Month 24. Primarily, reduction of the EDSS score had a positive impact on the better HRQoL outcomes, especially physical and social outcomes.

## Methods

The Lithuanian Bioethics Committee approved the study in 2011 (2011-01-27 No.: L-12-01/2), the permission to continue the study was granted by the Lithuanian Bioethics Committee in 2018 (2018-02-22 No.: 6B-18-41). All methods were carried out in accordance with relevant guidelines and regulations. All participants provided written informed consent. The AHSCT procedure is performed at our hospital as routine clinical practice for highly active relapsing patients with MS who do not respond to the second line MS treatment. Highly active MS patients in the case of AHSCT were defined the patients who experienced at least two relapses and had disability progression of at least 2.0 points according EDSS in the last year.

Participants underwent peripheral blood stem cells mobilization: cyclophosphamide was administered, subcutaneous filgrastim was started on day + 7 and peripheral blood stem cell apheresis procedure were targeted on day + 12 after cyclophosphamide. Collected cells were cryopreserved and stored. The conditioning regimen was intravenous cyclophosphamide and rabbit antithymocyte globulin^29^.

### Patient eligibility

The inclusion criteria were: diagnosis of highly active relapsing MS; aged 18 years and over; performed AHSCT and completed SF-36, Hospital Anxiety and Depression Scale (HADS), and Fatigue Descriptive Scale (FDS) questionnaires before AHSCT and during the period defined by the evaluation, and participants willing to cooperate voluntarily with the research.

### Neurologic assessment

Neurologic assessment was scheduled for all patients at baseline, Month 3, 12 and 24 after AHSCT. Neurological disability was assessed with EDSS^33^. Confirmed significant disability change was defined as disability increase from study baseline, measured by EDSS: increase of ≥ 1.0 points if baseline EDSS was ≤ 5.5 points or an ≥ 0.5-point increase if baseline EDSS was > 5.5 points.

### QoL

Eighteen patients in the study completed the SF-36 at baseline, Month 3, 12, and 24 after AHSCT. The SF-36 is a well-known, generic health-related QoL questionnaire with 36 items; it is one of the most used questionnaires assessing subjective psychological well-being in different disorders globally. Psychometric analysis of the translated versions shows that SF-36 is a reliable and valid measure in different languages and for different populations. It measures eight domains of health status: physical functioning, physical role limitations, bodily pain, general health perceptions, energy/vitality, social functioning, emotional role limitations, and mental health^34,35^. The scores are transformed to range from zero, indicating the worst possible health, to 100, indicating the best possible health.

### Anxiety and depression assessment

HADS was used to assess anxiety and depression. It was completed at baseline, Month 3, 12, and 24 after AHSCT. HADS is a brief self-administrated two-dimensional questionnaire, which is one of the most widely used tool to screen anxiety and depression among patients with different disorders^36^. Previous studies have shown that HADS is an appropriate screening instrument for patients with MS^37–39^. Of the 14 questions—seven reflect anxiety (Hospital Anxiety and Depression Scale—Anxiety [HADA subscale]) whereas the rest reflect depression (Hospital Anxiety and Depression Scale—Depression (HADD subscale)). A total subscale score of > 8 points out of a possible 21 indicate considerable symptoms of depression or anxiety.

### Fatigue assessment

The FDS was used for fatigue assessment in patients with MS at baseline and at Month 3, 12, and 24 after AHSCT. FDS is a valid, quick, and easy to conduct tool to evaluate the severity and quality of fatigue in a group of patients suffering from MS^40^. Lower scores indicate less fatigue-related disability.

### Statistical analysis

Data were analysed using statistical software package SPSS (version 20.0 for Windows). Outcomes are reported based on the last follow-up (Month 24) of each visit. Continuous variables were reported as medians and ranges or means and standard deviations, whereas categorical variables were reported as absolute numbers and percent of total patients. General linear model (GLM) repeated measures were used when assessing the data of different domains of QoL, fatigue, anxiety, and depression at different time points (baseline, Month 3, Month 12 and Month 24). To determine the significance of pairwise comparisons in one-way ANOVA, Bonferroni's post hoc test (for equal variances, Levene's test *p* > 0.05) was used. Mauchly's test of sphericity was used to test whether or not the assumption of sphericity was met in a repeated measures ANOVA. Linear regression was used to assess the impact of different demographic and clinical factors on the disability regression. A significance level *p* < 0.05 was accepted.

## Data Availability

The datasets used and/or analysed during the current study available from the corresponding author on reasonable request.
